# Hepatocyte Growth Factor-Induced Tendon Stem Cell Conditioned Medium Promotes Healing of Injured Achilles Tendon

**DOI:** 10.3389/fcell.2021.654084

**Published:** 2021-04-07

**Authors:** Zenan Zhang, Yutian Li, Tingting Zhang, Manyu Shi, Xin Song, Shulong Yang, Hengchen Liu, Mingzhao Zhang, Qingbo Cui, Zhaozhu Li

**Affiliations:** Department of Pediatric Surgery, The Second Affiliated Hospital of Harbin Medical University, Harbin, China

**Keywords:** tendon stem cell, hepatocyte growth factor, conditioned medium, paracrine, acellular therapy, tendon repair

## Abstract

Tendon repair is a medical challenge. Our present study investigated the effectiveness of acellular therapy consisting of conditioned medium (CM) of tendon stem cells (TSCs) induced with hepatocyte growth factor (HGF) in promoting the healing of injured Achilles tendon in a rat model. Proteomic analysis of soluble substances in the CM was performed using an array chip, and bioinformatic analysis was carried out to evaluate interactions among the factors. The effects of CM on viability and migratory capacity of tendon fibroblasts derived from rats with ruptured Achilles tendon were evaluated with the Cell Counting Kit 8 and wound healing assay, respectively. The expression of extracellular matrix (ECM)-related protein was assessed by western blotting. Rats with Achilles tendon injury were treated with CM by local injection for 2 weeks, and the organization of tendon fibers at the lesion site was evaluated by hematoxylin and eosin and Masson’s trichrome staining of tissue samples. The deposition and degradation of ECM proteins and the expression of inflammatory factors at the lesion site were evaluated by immunohistochemistry and immunofluorescence. Biomechanical testing was carried out on the injured tendons to assess functional recovery. There were 12 bioactive molecules in the CM, with HGF as the hub of the protein–protein interaction network. CM treatment enhanced the viability and migration of tendon fibroblasts, altered the expression of ECM proteins, promoted the organization of tendon fibers, suppressed inflammation and improved the biomechanics of the injured Achilles tendon. These results suggest that HGF stimulates the secretion of soluble secretory products by TSCs and CM promotes the repair and functional recovery of ruptured Achilles tendon. Thus, HGF-induced TSC CM has therapeutic potential for the treatment of tendinopathy.

## Introduction

Tendons are prone to injury through tearing or rupture as they are overstretched during physical activity (Kannus and Natri, 1997). Because of the inefficiency of the natural healing process, the formation of scar tissue is almost inevitable (Rhatomy et al., 2020). Moreover, spontaneously recovered tendons often exhibit substandard biomechanical properties and are susceptible to reinjury (Gaspar et al., 2015). Thus, unqualified repair can lead to long-term pain, discomfort, mobility impairment, and disability (Olsson et al., 2011; Gaspar et al., 2015; Braunstein et al., 2018). Various physical and biological interventions have been developed to improve the healing of injured tendons. For example, many stem cell (SC) types including mesenchymal (M)SCs derived from bone marrow (i.e., BMSCs) (Sharma and Snedeker, 2010; Yin et al., 2016; Veronesi et al., 2017), adipose tissue (Lee et al., 2017) and umbilical cord (Marmotti et al., 2018) were found to be effective in promoting tendon repair owing to their self-renewal capacity and multidifferentiation potential.

Stem cell-based treatments have certain limitations such as the short survival and tumorigenic potential of transplanted cells, local swelling, and undesirable spontaneous differentiation. Acellular therapies have been used to minimize these risks. Cultured MSCs promoted tissue regeneration through a paracrine mechanism involving the secretion of various factors (Vizoso et al., 2017). The resultant conditioned medium (CM) constituted a microenvironment that was conducive to the growth of SCs and contained soluble proteins, bioactive molecules, growth factors, and extracellular vesicles (EVs) or exosomes (Pawitan, 2014). BMSC CM was shown to promote tenocyte proliferation and reduce the levels of pro-inflammatory factors (Shimode et al., 2007; Chen et al., 2018). The effectiveness of BMSC-derived molecules in promoting tendon repair has been confirmed by other studies (Gissi et al., 2020; Yu et al., 2020). Tendon (T)SCs are a type of MSC found in tendon fascicles (Bi et al., 2007). Because of their strong tendency to differentiate into tenocytes (Zhang and Wang, 2010) and their secretion of trophic factors (Fu et al., 2017), TSCs have been used extensively in tendon repair and regeneration. Although there are some researches based on CM of TSCs, few studies to date have systematically analyzed the soluble components of TSC CM.

Conditioned medium can be modified to achieve a desired effect. Inflammation-stimulated or interferon γ-primed SC-derived EVs were shown to attenuate inflammation (Harting et al., 2018; Shen et al., 2020). It is possible that TSC CM can be similarly primed by specific biomolecules. In order to make the engineered TSC CM have the effect of promoting tissue healing, hepatocyte growth factor (HGF) can be used as an inducer. HGF, a typical paracrine growth factor, is mainly secreted by mesenchymal cells (Fukushima et al., 2018). It had an antifibrotic effect (Moon et al., 2019; Oka et al., 2019; Xie et al., 2019; Ma et al., 2020) and positive effects on tissue regeneration (Boldyreva et al., 2019; Choi J.S. et al., 2019; Choi W. et al., 2019) together with angiogenesis (Beilmann et al., 2004). Besides, it was thought to be a key factor in maintaining the stemness of hBMSCs (Cao et al., 2020) and MSC-EVs that encapsulated with HGF enhanced the barrier function of microvascular endothelial cells (Wang et al., 2017).

We speculate that CM of TSCs induced with HGF can promote tissue repair and functional recovery of a ruptured tendon. We tested our hypothesis with tendon fibroblasts for *in vitro* experiments and a rat model of Achilles tendon injury for *in vivo* investigations. Our results demonstrate that HGF-induced TSC CM is a novel acellular therapy that can facilitate the healing of injured tendon.

## Materials and Methods

### Isolation and Culture of TSCs

Tendon stem cells were isolated from Sprague-Dawley rats and cultured according to procedures described in our previous study (Zhang et al., 2020). Achilles tendons that were free of peritendinous tissues were harvested from euthanized rats. To reduce blood contamination, the samples were thoroughly washed with sterile phosphate-buffered saline (PBS) (Solarbio, Beijing, China). After digestion with trypsin (Beyotime, Shanghai, China), the specimens were cut into 1-mm^3^ blocks and were immersed in low-glucose Dulbecco’s Modified Eagle’s Medium (LG-DMEM) (Gibco, Grand Island, NY, United States) containing 3% w/v collagenase type I (Invitrogen, Carlsbad, CA, United States) and 4% w/v neutral proteinase (PeproTech, Rocky Hill, NJ, United States). The samples were incubated with gentle horizontal shaking for 1.5 h at 37°C, then passed through a 70-μm filter to obtain a single-cell suspension. After centrifugation, the cell pellet was resuspended in complete medium composed of LG-DMEM, fetal bovine serum (Biological Industries, Beit HaEmek, Israel) and penicillin plus streptomycin (Beyotime). The primary cells were cultured in T25 flasks in a humid environment with 5% CO_2_ at 37°C. The medium was changed for the first time on day 5 and replaced every 2 or 3 days thereafter. When they reached 80% confluence, the attached cells were released by trypsinization, identified and expanded for use in experiments.

### Preparation of CM

The following six TSC CM were prepared: CM1, LG-DMEM only; CM2, LG-DMEM with TSCs; CM3, LG-DMEM with 10 ng/ml HGF (PeproTech) + TSCs; CM4, LG-DMEM with 20 ng/ml HGF + TSCs; CM5, LG-DMEM with 40 ng/ml HGF + TSCs; and CM6, LG-DMEM with 80 ng/ml HGF + TSCs. The same number of TSCs were used in each preparation. After 24 h of culture, the supernatant from CM2–6 was collected and centrifuged, and the new supernatant was filtered to remove cell debris. The filtrate was divided into 2 equal parts. One portion was used in *in vitro* experiments and the other was concentrated using 3-kDa ultrafiltration centrifuge tubes (Millipore, Billerica, MA, United States) at 6000 × *g* for 1 h at 4°C for *in vivo* studies.

### Proteome Profiler Array and Bioinformatic Analysis

Based on cytokine antibody chip technology, the proteomic analyses of the soluble components of CM2–6 were conducted. All experimental procedures referred to the instruction of a Rat XL Cytokine Array Kit (R&D Systems, Minneapolis, MN, United States). A 1.5 ml solution containing 1.0 ml CM (i.e., the preparations described in section “Preparation of CM”) was dropped onto the surface of a blocked array chip. After overnight incubation at 4°C, the array chip was rinsed with washing buffer and then incubated with 1.5 ml diluted Detection Antibody Cocktail for 1 h at room temperature with gentle shaking. After washing, the array was incubated with horseradish peroxidase (HRP)–streptavidin for 30 min. Immunoreactive spots were detected with the Chemi Reagent Mix and the soluble secretory products of TSC were identified. By comparing the gray values of the corresponding spots on different array chips, the regulatory effect of HGF on the paracrine of TSC was verified. The experimental results were analyzed by cluster analysis and a protein–protein interaction (PPI) network was constructed using STRING v11.0^1^. The protein pairs with parameters of interaction relationships greater than 0.7 were listed.

### Ruptured Achilles Tendon Model

Male Sprague-Dawley rats weighing 160–180 g were purchased from the Laboratory Animal Center of Harbin Medical University. Animal experiments were carried out in accordance with the National Institutes of Health (NIH) Guide for the Care and Use of Laboratory Animals, and were approved by the Ethics Committee of Harbin Medical University. Proper biosafety measurements were also adopted.

To establish the complete Achilles tendon rupture model, amputation was performed at the midpoint of the calcaneal insertion and musculotendinous junction with a sharp surgical knife. The injured tendon was reconstructed by double-cross suturing with a 4-0 Vicryl absorbable suture (Johnson & Johnson, New Brunswick, NJ, United States) under a microscope. The skin incision was closed and reinforced with biological tissue glue (B. Braun, Melsungen, Germany). The rats were freely fed with food and water after they recovered from anesthesia. After 2 weeks, the naturally healed tendons of six rats were harvested for the isolation of tendon fibroblasts and all the other 63 rats used for *in vivo* experiments were sacrificed for sample harvest. Fifty one of the 63 rats were treated with CM2-6 (Group A–E). The condensed CM (0.1 ml) was postoperatively administered by local injection three times per week for 14 days. The other 12 rats (Group F) were left untreated as a control. The specific animal amount for each experiment was preciously introduced below.

### Isolation and Culture of Tendon Fibroblasts

The harvested naturally healed tendons were rinsed with cold sterile PBS. After mincing the tissue into 1- to 2-mm^3^ pieces, an explant culture was established to obtain primary cells. The composition of the culture medium and schedule for medium changes were the same as described for TSC culture. Third-generation long fusiform cells were used for *in vitro* experiments.

### Cell Counting Kit (CCK)-8

Tendon fibroblasts (1 × 10^4^) were seeded in a 96-well plate. After overnight incubation, the culture medium was replaced with 100 μl of CM1–6. The effect of CM on cell viability was evaluated with the CCK-8 assay (Dojindo, Kumamoto, Japan) according to the manufacturer’s instructions. The absorbance at 450 nm was measured at five time points (2, 4, 8, 12, and 24 h). The experiment was performed in triplicate and the data were averaged. Results for groups treated with CM2–6 are presented relative to the values for the CM1-treated group.

### Wound Healing Assay

As in our previous study (Han et al., 2019), tendon fibroblasts were cultured in 6-well plates. When the cells reached 100% confluence, a sterile 200-μl pipette tip and ruler were used to draw a smooth scratch mark across the bottom of each well. After washing off the scraped cells with sterile PBS, 2 ml CM was added to each well. Photos were obtained with an optical microscope (Olympus, Tokyo, Japan) at five time points (0, 12, 24, 48, and 60 h). The cell migration rate at each time point was calculated based on the corresponding acellular zone at 0 h.

### Western Blotting

Tendon fibroblasts were cultured with CM for 24 h, then lysed in precooled radioimmunoprecipitation assay buffer (Solarbio) supplemented with phenylmethylsulfonyl fluoride (Beyotime) for 30 min. Cell debris was cleared by centrifugation at 12,000 × *g* for 20 min at 4°C. The total protein concentration was determined with the BCA Protein Assay Reagent kit (Beyotime). The protein samples were diluted with 5 × sodium dodecyl sulfate loading buffer (Beyotime) and 20 μg protein per lane was electrophoretically separated on a 10% polyacrylamide gel (Beyotime). The proteins were transferred to polyvinylidene difluoride membranes and blocked with 5% non-fat dry milk (BD Biosciences, Franklin Lakes, NJ, United States) for 2 h at room temperature. Then, the membranes were separately probed overnight at 4°C with primary antibodies against collagen (COL)III (1:500), fibronectin (1:2000), matrix metalloproteinase (MMP)-1 (1:2000), MMP-9 (1:500) (all from Abcam, Cambridge, United Kingdom) and β-actin (1:1000) (WanleiBio, Shenyang, China). The membranes were rinsed with Tris-buffered saline (Solarbio) with 0.1% Tween-20 (Solarbio) and then incubated with the appropriate HRP-conjugated secondary antibodies (Boster Bio, Wuhan, China) for 1.5 h at room temperature. Protein bands were visualized using enhanced chemiluminescence substrate reagent (HaiGene, Harbin, China). The gray value was quantified with ImageJ software (NIH, Bethesda, MD, United States).

### Immunohistochemistry Analysis

Extracellular matrix (ECM) organization at the tendon lesion site following CM treatment was evaluated by immunohistochemistry. Tendon specimens (1 cm long) harvested from 15 CM treated rats (*n* = 3) and 3 untreated ones were immediately immersed in 4% paraformaldehyde solution (BioSharp, Hefei, China). After dehydration through a graded alcohol series, samples were embedded in paraffin and serial sections of the tissue blocks were cut at a thickness of 5 μm parallel to the long axis of the tendons on a microtome (Leica, Wetzlar, Germany). After antigen retrieval, tissue sections mounted on glass slides were treated with 3% hydrogen peroxide to quench endogenous peroxidase activity, blocked with 10% goat serum (Solarbio) for 15 min at room temperature and incubated overnight at 4°C with primary antibodies against COLIII (1:100), fibronectin (1:100), α-smooth muscle actin (SMA) (1:100), MMP-2 (1:100), MMP-9 (1:100), tissue inhibitor of metalloproteinase (TIMP)-1 (1:100), and vascular endothelial growth factor (VEGF) (1:100) (all from Abcam). The slides were then incubated in the dark with species-specific secondary antibodies. Diaminobenzidine (Solarbio) was used to visualize the bound antibody complex. After dehydrating the sections and covering the slides with glass coverslips, the sections were examined and photographed under a microscope (Olympus) mounted with a camera. Positive signals were quantified with ImageJ software.

### Histological Analysis

Tendon samples were harvested from 15 CM treated rats (*n* = 3). Normal tendons were also collected. After dehydration and embedding in paraffin, the samples were also cut into 5-mm thickness serial sections. Then, the slices were stained with hematoxylin and eosin (H&E) and Masson’s trichrome and observed under a light microscope. The organization of tendon fibers was evaluated according to a previously described parallel fiber alignment scoring method (Jiang et al., 2016) by investigators who were blinded to group assignment. The scoring scale was as follows: 0, 0–25% parallel fiber alignment; 1, 25–50%; 2, 50–75%; and 3, 75%–100%.

### Immunofluorescence Analysis

The expression of the pro-inflammatory cytokine interleukin (IL)-6 and anti-inflammatory cytokine IL-10 was detected by immunofluorescence analysis. The sample collection was same as the description in section “Immunohistochemistry analysis”. Tendon tissue samples were embedded in optimal cutting temperature compound and frozen serial sections were cut at a thickness of 10 μm. After antigen retrieval, the sections were blocked with 10% goat serum for 15 min at room temperature, then incubated overnight at 4°C with primary antibodies against IL-6 (1:100) and IL-10 (1:100) (both from Abcam), followed by Cy3-conjugated goat anti-rabbit IgG (Beyotime) for 60 min at room temperature. Nuclei were stained with DAPI (Beyotime). Images were acquired under a fluorescence microscope (Olympus), and the area of positive signal was determined using ImageJ software.

### Biomechanical Testing

We evaluated the effectiveness of the CM with the greatest therapeutic effect in all the above experiments [sections “Cell Counting Kit (CCK)-8,” “Wound healing assay,” “Western blotting,” “Immunohistochemistry analysis,” “Histological analysis,” and “Immunofluorescence analysis”] in promoting functional recovery in the rat model of Achilles tendon rupture. Eighteen tendon-calcaneal complexes were carefully dissected from six CM treated rats, six naturally healed rats, and six intact animals (i.e., without injury). They were used for biomechanical testing as previously described (Chamberlain et al., 2019). Briefly, the transverse diameter (TD) and anteroposterior diameter (AD) of the healed sections were measured with electronic vernier calipers. The tissue sample’s cross-sectional area (S) was approximated as an oval, and was calculated with the equation S = πTD × AD/4. A universal tensile testing machine (Hengruijin, Jinan, China) was used for testing. The proximal muscle wrapped in a piece of 100 × sandpaper and the distal naked paw were affixed to the two testing clamps. An axial 0.1 N preload was applied along the long axis of the tendon, with elongation at a speed of 5 mm/min. The maximum tensile load was recorded. Stiffness and Young’s modulus were calculated based on the linear slope of the strain–stress curve.

### Statistical Analysis

Data are presented as mean ± SD. Differences between groups were evaluated by one-way analysis of variance (ANOVA) followed by Tukey’s *post hoc* test if necessary or by two-way ANOVA using Prism v8.3.0 software (GraphPad, La Jolla, CA, United States). *P* < 0.05 was considered statistically significant.

## Results

### Characterization of TSCs

**FIGURE 1:**
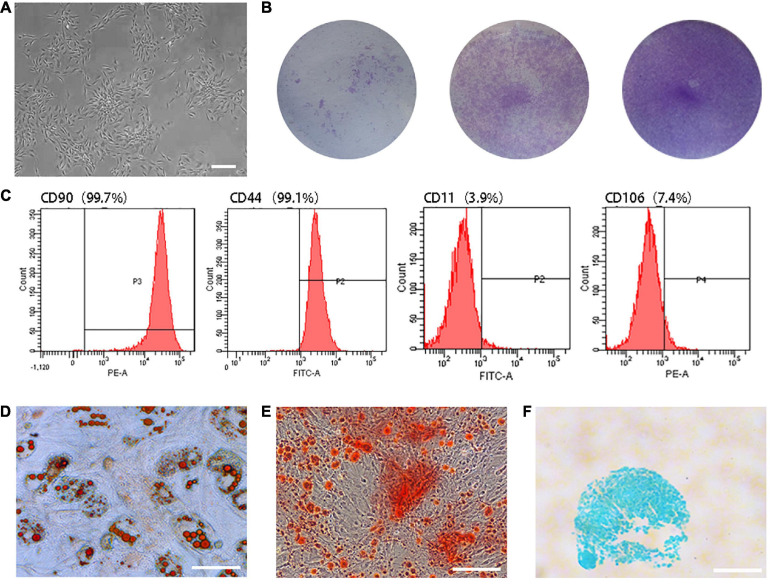
Characterization of TSCs. **(A)** Primary TSCs were spindle-shaped and reached 40–50% confluence on Day 10 after seeding. **(B)** After 7 days of culture, cell colonies were visualized by crystal violet staining. The initial seeding densities were 1 × 10^3^, 1 × 10^4^, and 1 × 10^5^ per well, respectively. **(C)** Flow cytometry analysis revealed that 99.7% of cells expressed CD90 and 99.1% expressed CD44. Meanwhile, only 3.9% and 7.4% expressed CD11 and CD106. **(D)** Lipid droplets were observed by oil red O staining after adipogenic differentiation induction of TSCs. **(E)** Mineralized nodules were observed by alizarin red staining after osteogenic differentiation induction of TSCs. **(F)** Acid mucopolysaccharide was observed by Alcian blue staining after chondrogenic differentiation induction of TSCs. All bars: 200 μm.

Primary TSCs were spindle-shaped, reaching 40–50% confluence on Day 10 after seeding (Figure 1A). The parts stained by crystal violet were the cell colonies (Figure 1B). Flow cytometry analysis revealed that 99.7% of cells expressed the cell surface marker cluster of differentiation (CD) 90 and 99.1% expressed CD44. Meanwhile, only 3.9% and 7.4% expressed CD11 and CD106, respectively (Figure 1C). TSCs had multi-differentiation potential, as determined by the detection of lipid droplets (Figure 1D), mineralized nodules (Figure 1E), and acid mucopolysaccharide (Figure 1F).

### Proteomic Analysis of CM and PPIs of Soluble Bioactive Molecules

**FIGURE 2:**
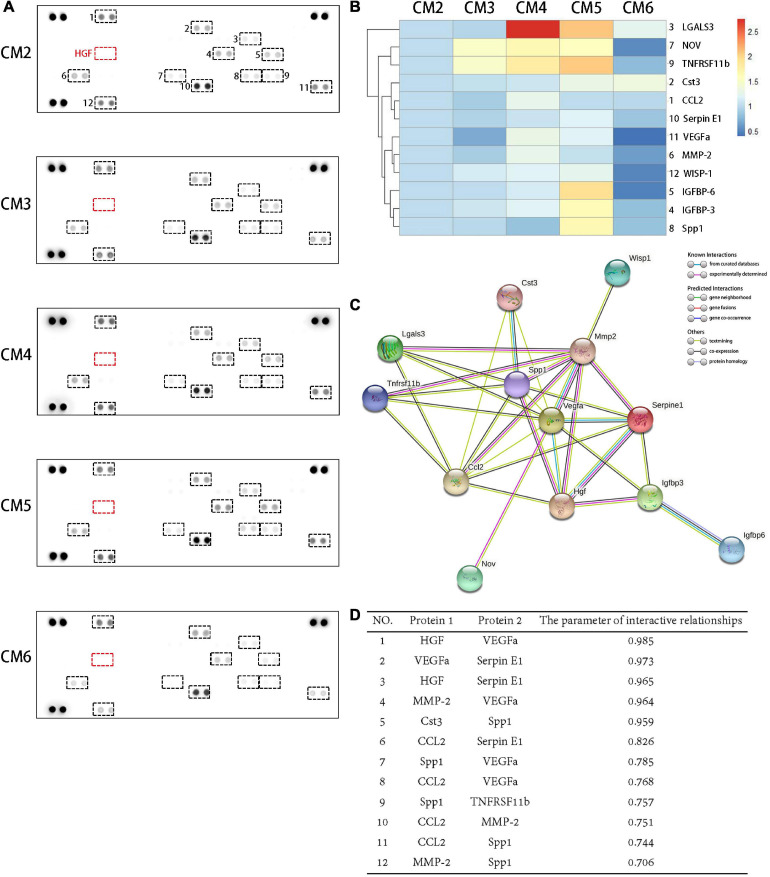
Proteomic analysis of CM and PPIs of soluble bioactive molecules. **(A)** A total of 12 molecules were detected in the CM2 with the proteome profiler antibody array. The concentrations of soluble bioactive molecules can be modified by HGF. **(B)** A cluster analysis result was shown by a heat map. Compared with CST3, CCL2, Serpin E1, VEGFa, MMP-2, and WISP-1, the concentration variations of LGALS3, NOV, and TNFRSF11b were sensitive to the dose of HGF. While, only when the concentration of HGF was 40 mg/ml, the contents of IGFRP-6, IGFBP-3, and SPP1 changed obviously. **(C)** PPIs network of the 12 proteins detected in TSC CM and HGF. Known interactions are indicated by light blue (weak) or purple (strong) lines. **(D)** Protein pairs with interaction score > 0.7. HGF and VEGFa, VEGFa and Serpin E1, HGF and Serpin E1, MMP-2 and VEGFa, CST3 and SPP1 showed strong interactions (>0.9).

A total of 12 molecules were detected in the CM2 with the proteome profiler antibody array. HGF contained in the CM3–6 were depleted from the culture medium after 24 h of culture. As a result, it caused variations in the concentrations of CM components but no generation of new products (Figure 2A). Almost all the secretion of these bioactive molecules can be inhibited by a high concentration of HGF (i.e., CM6 with 80 ng/ml HGF). Compared with CST3, CCL2, Serpin E1, VEGFa, MMP-2, and WISP-1, the concentration variations of LGALS3, NOV, and TNFRSF11b were sensitive to the dose of HGF. While, only when the concentration of HGF was 40 mg/ml, the contents of IGFRP-6, IGFBP-3, and SPP1 changed obviously (Figure 2B). PPIs of the 12 molecules are shown in Figure 2C. HGF interacted with 6 proteins. SPP1, CCL2, and MMP-2 interacted with eight proteins. In maximum, there were 10 PPIs for VEGFa. WISP-1 only interacted with MMP-2 but not with other proteins; the same was true for IGFBP-6 and IGFBP-3, NOV and VEGFa. The strength of the interactions are shown in Figure 2D. LGALS3, IGFBP-3, IGFBP-6, NOV, and WISP-1 had a low degree of interaction (<0.7), whereas HGF and VEGFa, VEGFa and Serpin E1, HGF and Serpin E1, MMP-2 and VEGFa, CST3 and SPP1 showed strong interactions (>0.9).

### CM Promotes the Viability and Migration of Tendon Fibroblasts

**FIGURE 3:**
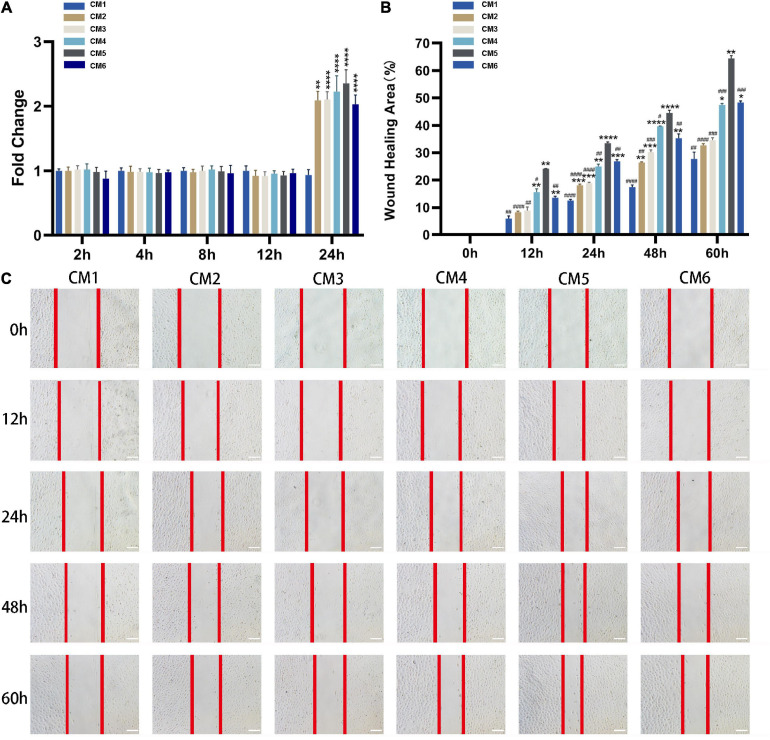
CM promotes the viability and migration of tendon fibroblasts. **(A)** Cell viability was evaluated with CCK-8. CM treatment had no effect on the viability of tendon fibroblasts at 2, 4, 8, and 12 h, but the viability was doubled at 24 h. The effect was independent of HGF concentration. **(B,C)** The migration of tendon fibroblasts was evaluated with the wound healing assay. Red lines indicate the migration front. The wound closure of the fibroblasts with CM1-5 increased gradually. The maximum rate of migration was observed with CM5. No further increase of wound closure was observed with CM6. Bars: 200 μm. *, versus CM1. * means *p* < 0.05, ** means *p* < 0.01, *** means *p* < 0.001, **** means *p* < 0.0001. #, versus CM5. # means *p* < 0.05, ## means *p* < 0.01, ### means *p* < 0.001, #### means *p* < 0.0001.

Conditioned medium treatment had no effect on the viability of tendon fibroblasts at 2, 4, 8, and 12 h, but the viability was doubled at 24 h. No statistical significance was observed among CM2-6 (Figure 3A). Likewise, CM accelerated the migration of tendon fibroblasts (Figures 3B,C). The effect showed a dose-dependent relationship with the concentration of HGF. Obviously, the wound closure of the fibroblasts with CM1-5 increased gradually. The maximum rate of migration was observed with CM5 (64.48% ± 0.80% wound closure after 60 h). When the concentration of HGF reached at 80 ng/ml (i.e., CM6), no further increase of wound closure was observed. In fact, it yielded a result similar to that obtained with CM4.

### CM Alters ECM Composition and Promotes Angiogenesis

**FIGURE 4:**
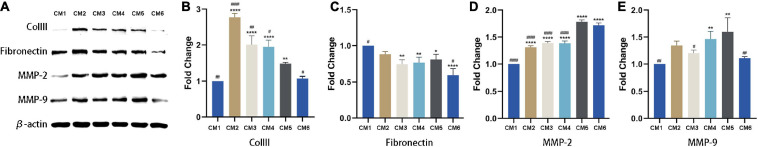
Expression of ECM-related factors detected by western blotting. **(A)** COLIII, fibronectin, MMP-2 and MMP-9 levels were detected by western blotting. β-actin served as the loading control. **(B,C)** Quantitative analysis result of COLIII and fibronectin. With the increase of HGF concentration, their expressions decreased. **(D,E)** Quantitative analysis result of MMP-2 and MMP-9. They were upregulated following the applications of CMs. *, versus CM1. * means *p* < 0.05, ** means *p* < 0.01, **** means *p* < 0.0001. #, versus CM5. # means *p* < 0.05, ## means *p* < 0.01, #### means *p* < 0.0001.

**FIGURE 5:**
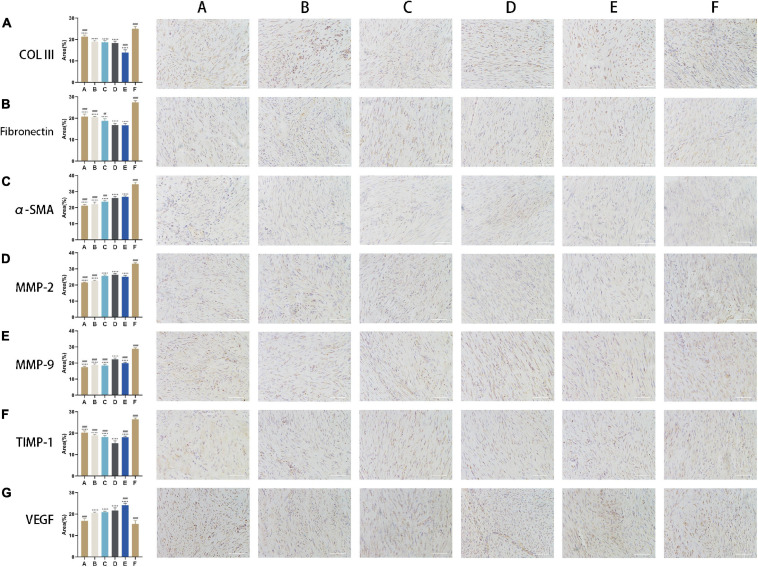
CM alters ECM composition and promotes angiogenesis. **(A,B)** Expression levels of COLIII and fibronectin. With the increase of HGF concentration, their expressions decreased. **(C)** Expression level of α-SMA. Its expression increased following the applications of CMs. **(D,E)** Expression levels of MMP-2 and MMP-9. With the increase of HGF concentration, their expressions increased. **(F)** Expression level of TIMP-1. Its expression first decreased and then increased. The turning point came at Group D. **(G)** Expression level of VEGF. It was upregulated in the presence of CM, with the highest level detected in cells treated with CM6. Bars: 200 μm. #, versus D. ## means *p* < 0.01, ### means *p* < 0.001, #### means *p* < 0.0001. +, versus F. ++++ means *p* < 0.0001.

*In vitro* experiment, MMP-2 and MMP-9 were upregulated following the application of CM (Figures 4A,D,E). The expression of MMP-2 was highest in tendon fibroblasts treated with CM5 and CM6 compared to the other CM formulations, with no difference between the two groups. Similar results were obtained for MMP-9, which showed the highest expression in the CM5-treated group. With the increase of HGF concentration, their expressions of COLIII and fibronectin decreased (Figures 4A–C). All the above results, upregulated MMPs and downregulated ECM components, were verified *in vivo* experiment by immunohistochemistry (Figures 5A,B,D,E). Although theirs levels were lower than that of Group F, the expression of α-SMA was increased among experimental groups treated with CM2-6 (Figure 5C). Meanwhile, the expression of TIMP-1 first decreased and then increased with increasing concentrations of HGF (Figure 5F). The turning point came at Group D. VEGF, which promotes angiogenesis, was upregulated in the presence of CM, with the highest level detected in tissues treated with CM6 (i.e., Group E) (Figure 5G).

### CM Promotes the Orderly Arrangement of Tendon Fibers

**FIGURE 6:**
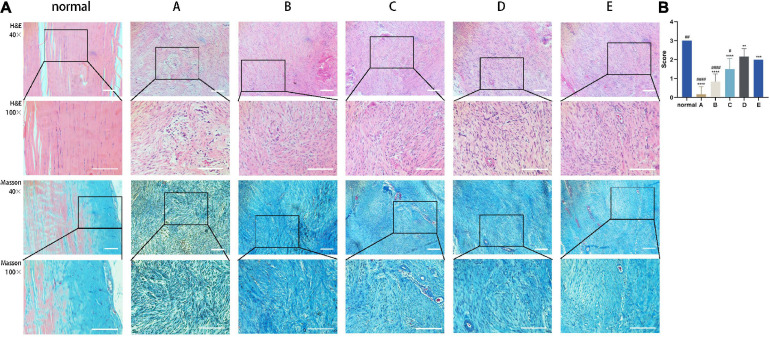
CM promotes the orderly arrangement of tendon fibers. **(A)** Sections of healed tendon tissue stained with H&E and Masson’s trichrome were observed by light microscopy at 40× and 100× magnification. Achilles tendon tissue sections treated with CM5 and CM6 (i.e., Group D and Group E) showed a much more regular and compact alignment of collagen fibers compared to the other groups. **(B)** Histogram of fiber alignment score. With the increase of HGF concentration, tendon fibers arranged orderly. Bars: 500 μm. ^∗^, versus normal. ^∗∗^ means *p* < 0.01, ^∗∗∗^ means *p* < 0.001, ^****^ means *p* < 0.0001. #, versus D. # means *p* < 0.05, ## means *p* < 0.01, #### means *p* < 0.0001.

The effect of CM on tendon fiber alignment was evaluated by histological analysis. Achilles tendon tissue sections treated with CM5 and CM6 (i.e., Group D and Group E) showed a much more regular and compact alignment of collagen fibers compared to those treated with CM2 and CM3 (i.e., Group A and Group B), which had disorganized fibers (Figure 6A). Samples treated with CM4 (i.e., Group C) had an intermediate phenotype. These results were also confirmed by the fiber alignment score, which was higher for samples treated with CM5 and CM6 than for the other three groups (Figure 6B).

### CM Suppresses Inflammation in Injured Tendon

**FIGURE 7:**
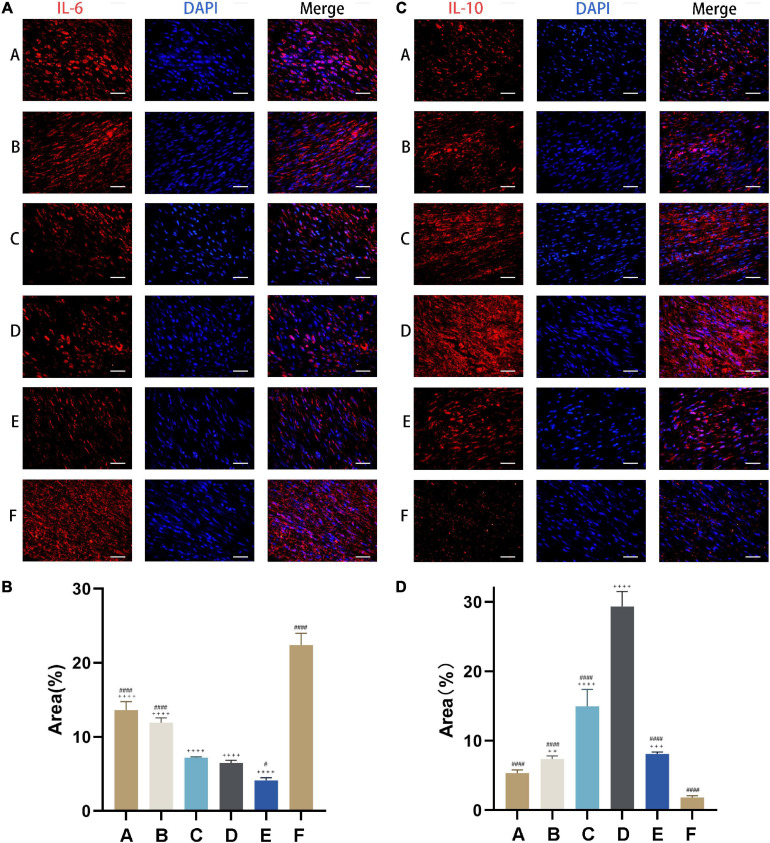
CM suppresses inflammation in injured tendon. **(A)** Expression of the pro-inflammatory cytokine IL-6 (red) was detected by immunocytochemistry. Nuclei were stained with DAPI (blue). CM held the capacity of inhibiting pro-inflammatory cytokine expression. **(B)** Quantitative analysis of IL-6 expression levels based on the area of positive signal. **(C)** Expression of the anti-inflammatory cytokine IL-10 (red) was detected by immunocytochemistry. Nuclei were stained with DAPI (blue). CM held the capacity of promoting anti-inflammatory cytokine expression. **(D)** Quantitative analysis of IL-10 expression levels based on the area of positive signal. Bars: 50 μm. #, versus D. # means *p* < 0.05, #### means *p* < 0.0001. +, versus F. ++ means *p* < 0.01, +++ means *p* < 0.001, ++++ means *p* < 0.0001.

IL-6 level was highest in rats with Achilles tendon injury that were left untreated (Group F) and decreased with CM treatment (Figure 7A). The capacities of inhibiting pro-inflammatory cytokine expression in Group A and Group B (i.e., treatments with CM2 and CM3) were weaker than those in Group C, Group D, and Group E. However, the difference between Group C and Group D was not statistically significant (Figure 7B). The opposite trend was observed for IL-10, which showed the lowest expression in Group F (Figure 7C). With the treatment of CM, anti-inflammatory cytokine expressions increased. The effect was greater at higher concentration of HGF. It maximized in Group D. When it came to Group E, no further increase of anti-inflammatory cytokine expression was observed. In contrast, it dropped to the same value as that in Group B (Figure 7D).

### CM Enhances the Biomechanical Properties of Injured Achilles Tendon

**FIGURE 8:**
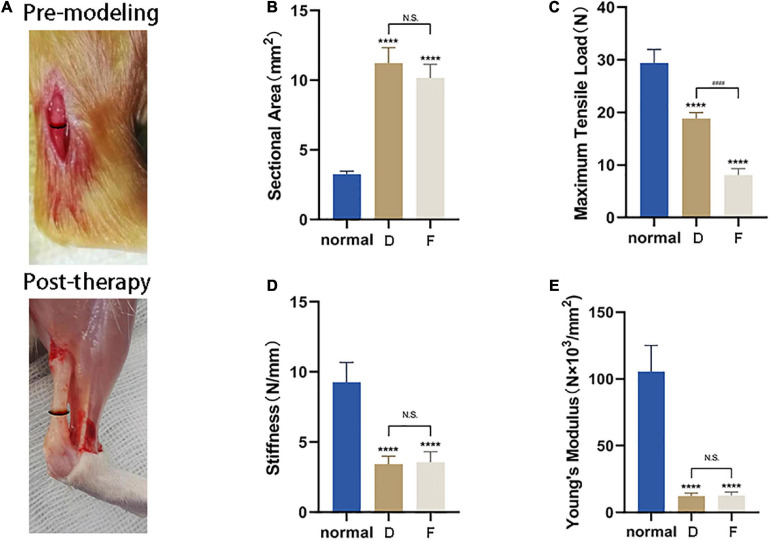
CM enhances the biomechanical properties of injured Achilles tendon. **(A)** Lesion area before and after tendon repair. **(B)** Healed tendon from Group D and F had a cross-sectional area nearly three times that of uninjured tissue. **(C)** After CM treatment, functional improvement of the maximum tensile load was still lower than in normal tendon, corresponding to 60% recovery. **(D)** The stiffness of samples in Group D and Group F were still far less than that of normal tissues. There were no differences between the two experimental groups. **(E)** Recovery of Young’s modulus of samples was same as that of stiffness. *, versus normal. **** means *p* < 0.0001; #, D versus F, #### means *p* < 0.0001.

Based on the above results, tendons from Group D (i.e., treatment with CM5) were used for biomechanical testing. Healed tendon from Group D and F had a cross-sectional area nearly three times that of uninjured tissue (Figures 8A,B). Although there was functional improvement after CM treatment, the maximum tensile load was still lower than normal tendon (Figure 8C), corresponding to 60% recovery. What was worse, naturally healed tendons in Group F only recovered 30% of the normal maximum tensile load. The stiffness and Young’s modulus of samples in Group D and Group F were still far less than that of normal tissues. There were also no differences between CM treated tendons and naturally healed ones (Figures 8D,E).

## Discussion

The results of our study confirmed the secretory function of TSCs: the CM produced after 24 h of culture contained more than 12 bioactive molecules, including those were known to promote tissue healing or exert anti-inflammatory and antifibrotic effects. Specifically, C–C motif chemokine ligand (CCL)2 stimulated macrophage chemotaxis. CST3 was shown to alleviate tissue fibrosis in kidney and lung (Kim et al., 2018a, b). LGALS3, also known as galectin-3, functioned as a regulatory molecule at various stages of inflammation from acute to chronic as well as in tissue fibrogenesis (Henderson and Sethi, 2009). MMP-2 was involved in ECM remodeling. SPP1 played an important role in the production of collagen and the resolution of inflammation (Ruberti et al., 2018; Bevan et al., 2020). Serpin E1 regulated tissue homeostasis and promoted wound healing by inhibiting plasmin-mediated MMP activation (Flevaris and Vaughan, 2017). VEGF mediated angiogenesis. Thus, the combined presence of these factors in TSC CM had a positive effect on tendon healing. Our results also showed that the levels of these factors in the CM varied with HGF concentration. Based on the results of the bioinformatic analysis, we inferred that HGF regulated the secretory activity of TSCs. The PPI network of secreted molecules revealed strong connections between HGF and VEGFa (0.985) and between HGF and Serpin E1 (0.965), which were consistent with previous reports (Chang et al., 2013; Su et al., 2013). HGF might stimulate the release of VEGFa and Serpin E1, which interacted with or activate the release of MMP-2, CCL2 and SPP1. SPP1 then induced the expression of tumor necrosis factor receptor superfamily member (TNFRSF)11b and CST3. Owing to the complexity of the PPIs involving HGF, the 5 TSC CMs (i.e., CM2-6) tested in our study had distinct therapeutic effects.

Tissue repair is a complex process (Leong et al., 2020) that consists of three stages: inflammation, proliferation, and remodeling (Thankam et al., 2018). Inflammation occurs in the early stage of tissue damage and can lead to increased vascular permeability, neovascularization and local recruitment of inflammatory cells. Suppressing this response is beneficial for the healing of injured tendons (Blomgran et al., 2017; Chamberlain et al., 2019; Shi et al., 2019). Macrophages play an important role in this process (Hays et al., 2008; Millar et al., 2017). M1- and M2-polarized macrophages respectively mediate inflammation and its resolution. The switch from the former to the latter stimulates tendon tissue repair (Manning et al., 2015). IL-6—a pro-inflammatory cytokine associated with macrophage activation (Munder et al., 1998)—was used as a marker in our study along with the anti-inflammatory cytokine IL-10, which could stimulate M2 polarization of macrophages. The ratio of M1- to M2-polarized macrophages depends on cues in the microenvironment (Gordon and Martinez, 2010). Upon treatment with TSC CM, IL-6 was downregulated and IL-10 was upregulated at the lesion site, suggesting that CM modulated the inflammatory response by inducing macrophage phenotype switching. Thus, like EVs or exosomes isolated from some SC types (Shen et al., 2020; Shi et al., 2019), TSC CM can also attenuate the early inflammatory response.

Rat BMSC CM and the secretome of human MSCs were shown to enhance the viability of tenocytes and stimulate cell migration (Chen et al., 2018; Sevivas et al., 2018). This is in agreement with our observations. Given that fibroblasts would fill with the lesion during tendon repair, tendon fibroblasts extracted from the defect site were used as the target cells in our *in vitro* experiments. The TSC CM enhanced cell viability at 24 h and promoted cell migration up to 60 h. Thus, local CM injection every 2 or 3 days may be an effective strategy to promote tendon repair.

Tissue repair involves a balance between ECM deposition and degradation. MMPs play an important role in the latter in cooperation with TIMPs. Ciprofloxacin induced MMP-2 expression in tendon (Tsai et al., 2011) while exosomes from TSCs inhibited the expression of MMP-3 and increased that of TIMP-3 (Wang et al., 2019). In our study, the expressions of MMP-2 and MMP-9 were gradually upregulated while TIMP-1 had an opposite tendency after the treatments with CM2-5, indicating that there was a balance in MMPs and TIMPs. Collagen (e.g., COLIII) and non-collagenous glycoproteins (e.g., fibronectin) are the main components of ECM. COLIII accounted for around 10% of the total collagen in tendon tissue and excessive amounts could result in scar formation (Tsai et al., 2006; Xu et al., 2018). With the increasing activity of MMP-2 and MMP-9 in each experimental group, the expression of COLIII and fibronectin was decreased gradually. This outcome benefited the injured tendon remodeling. α-SMA was a marker of myofibroblasts, which were derived from activated fibroblasts (Sun et al., 2019). It has a pair of opposite physiology effects. The negative side, excessive myofibroblasts lead to scar formation. The expressions of α-SMA in CM treated groups were much lower than that in control group, indicating that CM can inhibit the formation of scar. The positive side, myofibroblasts contribute to wound contraction. Our results showed that the level of α-SMA in experimental groups was increased along with the increase of HGF concentration. That is to say that higher dose of HGF posts more obvious effect on tissue repair. In addition, early angiogenesis promoted tissue repair (Chamberlain et al., 2019); VEGF, a marker of angiogenesis, was upregulated at the lesion site following treatment with CM, which likely contributed to tendon repair in our model.

The ultimate goal of tendon healing is functional restoration, as determined by the ability to resume normal activities without experiencing pain or recurrence of the injury. Load bearing by the Achilles tendon in rat was increased by TSC exosome injection (Wang et al., 2019); and stiffness and Young’s modulus were improved following application of EV-educated macrophages (Chamberlain et al., 2019). We found that the maximum tensile load that could be borne by healed Achilles tendon was increased by CM5 treatment. This was in line with our observation that the tendon tissue of CM5-treated rats had a much more parallel and dense arrangement of fibers. There are two possible explanations for why there were no improvements in stiffness and Young’s modulus in our study. The change in stress may not have adapted to the rapid tendon displacement—that is, the slope of the strain–stress curve is not sufficiently large. Alternatively, the enlargement of the cross-sectional area of the healed lesion site may have dispersed the tensile force. Therefore, rehabilitation training should be carried out reasonably after treatment with HGF-induced TSC CM. Meanwhile, there is a risk that the healed Achilles tendon could re-rupture.

The results of our study demonstrate that the therapeutic effects of TSC CM varied with HGF concentration; 40 ng/ml HGF showed maximal effectiveness in terms of stimulating cell migration, modulating ECM and inflammation, and restoring tendon fiber alignment and biomechanical properties. A limitation of our study is that we do not carry out long-term observations of tissue remodeling following CM treatment. Additionally, a more detailed investigation of the mechanisms underlying the therapeutic effects of CM is warranted. Whether the therapeutic effect of HGF-induced TSC CM is mediated by exosome needs a further study. Nonetheless, HGF-induced TSC CM treatment enhanced the viability and migration of tendon fibroblasts, altered the expression of ECM proteins, promoted the organization of tendon fibers, suppressed inflammation and improved the biomechanics of the injured Achilles tendon.

## Conclusion

These results suggest that HGF stimulates the secretion of soluble secretory products by TSCs and CM promotes the repair and functional recovery of ruptured Achilles tendon. Thus, HGF-induced TSC CM has therapeutic potential for the treatment of tendinopathy.

## Data Availability Statement

The original contributions presented in the study are included in the article. Further inquiries can be directed to the corresponding author.

## Ethics Statement

The animal study was reviewed and approved by the Ethics Committee of Harbin Medical University.

## Author Contributions

ZZ and YL contributed equally to this work and shared first authorship. ZZ, QC, and ZL conceived this study. ZZ, YL, TZ, and XS conducted all the experiments and data analyses. MS participated in data curation and statistical analysis. SY, HL, and MZ provided the experimental technical guidance. ZZ finished the original draft. YL, QC, and ZL gave text revision and edition. All the authors read the final manuscript.

## Conflict of Interest

The authors declare that the research was conducted in the absence of any commercial or financial relationships that could be construed as a potential conflict of interest.

## Footnotes

1 https://string-db.org/
